# 基于内部萃取电喷雾电离质谱法的不同产地生姜主要差异成分分析

**DOI:** 10.3724/SP.J.1123.2025.09001

**Published:** 2026-08-08

**Authors:** Huiting WANG, Siyu XIE, Jingjing XIE, Ruotian WU, Manman QIN, Huanwen CHEN

**Affiliations:** 1. 江西中医药大学，恶性肿瘤中医药诊疗与康复江西省重点实验室，江西 南昌 330004; 1. Key Laboratory for Diagnosis，Treatment，and Rehabilitation of Cancer using Chinese Medicine，Jiangxi University of Chinese Medicine，Nanchang 330004，China; 2. 河南中医药大学第三附属医院药学部，河南 郑州 450000; 2. Department of Pharmacy，the Third Affiliated Hospital of Henan University of Chinese Medicine，Zhengzhou 450000，China

**Keywords:** 生姜, 内部萃取电喷雾电离质谱, 差异成分, 产地, ginger, internal extractive electrospray ionization mass spectrometry （iEESI-MS）, differential components, origins

## Abstract

生姜为姜科植物姜*Zingiber officinale* Rosc*.*的新鲜根茎，产地是影响生姜质量的关键因素，然而现有分析方法往往耗时费力。为实现不同产地生姜的主要化学成分和差异成分的快速定性定量分析，本研究选取云南、四川、贵州及河南4个产地的生姜样本，采用内部萃取电喷雾电离质谱（iEESI-MS）结合多元统计方法，建立了一种快速鉴定不同产地生姜中27种化学成分和定量分析3种主要差异成分的方法。称取5.0 mg生姜碎片放入自制iEESI-MS装置上的滤膜中，在优化萃取剂、离子源喷雾口到质谱口的距离、喷雾电压、离子传输管温度等参数后，得到不同产地生姜的一级质谱数据，通过主成分分析与偏最小二乘判别分析模型实现了不同产地生姜的清晰区分，并结合生姜主要成分和差异成分的相关性分析筛选出6-姜酚、8-姜酚和L-丝氨酸3种标志性差异成分。对该3种成分的定量分析显示，各成分在2.0~20 000.0 μg/g范围内线性良好（决定系数*R*²≥0.996），方法灵敏度高（检出限为3.0~20.0 μg/g，定量限为10.0~50.0 μg/g），回收率为98.8%~100.9%，相对标准偏差（RSD）≤1.6%。利用所建立的方法对不同产地生姜进行检测，并结合热图分析进一步揭示了产地与主要差异成分含量之间的关联性。本研究为生姜质量的快速评价与产地鉴别提供了可靠平台，具有重要的应用价值。

## Abstract (en)

Ginger， the fresh rhizome of *Zingiber officinale* Rosc. from the Zingiberaceae family， is highly valued worldwide for its unique aromatic profile and distinct pungent flavor. It is widely utilized both as a culinary spice and a natural food additive. Globally， ginger comprises 53 known genes and approximately 1 300 varieties， predominantly cultivated in tropical and subtropical regions. Major producing countries include India， China， Indonesia， and Nigeria， with China ranking as the second-largest producer. In many Asian countries， such as China， Korea， and Japan， ginger has long been recognized not only for its culinary applications but also for its medicinal properties. It is traditionally employed to alleviate cold symptoms， promote body warmth， reduce nausea， suppress coughs by clearing phlegm， and even neutralize toxins from seafood consumption. The efficacy and safety of ginger as a medicinal herb are largely determined by the content and purity of its active constituents， such as gingerols and shogaols. Thus， accurate quality assessment is essential to ensure its therapeutic value. Moreover， the contents of these bioactive compounds can vary considerably depending on the geographical origin. In China， high-quality medicinal ginger is primarily cultivated in provinces such as Yunnan， Sichuan， Guizhou and Henan. Given the complexity and diversity of ginger’s chemical composition， it is crucial to understand its characteristic components and their variation with origin. This knowledge supports not only accurate quality evaluation but also reliable traceability of ginger sources. Ultimately， it facilitates the selection of ginger with superior medicinal properties for direct use in traditional medicine or as high-quality raw material in pharmaceutical development. However， conventional analytical techniques for determining origin and quality， such as high performance liquid chromatography （HPLC） and gas chromatography-mass spectrometry （GC-MS）， are often time-consuming， require extensive sample preparation， and consume large volumes of solvents. To overcome these limitations， this study developed a high-throughput method based on internal extractive electrospray ionization mass spectrometry （iEESI-MS） for rapid compositional profiling and origin authentication of ginger. Samples were collected from four major ginger-producing regions in China： Yunnan， Sichuan， Guizhou， and Henan. Each sample was minimally processed into fragments， with only 5.0 mg used for analysis. The fragments were placed on a filter membrane inside a custom-built iEESI-MS device. Key instrumental parameters such as the extraction solvent composition， ion transfer tube temperature， spray voltage， and solvent flow rate were systematically optimized to enhance detection sensitivity and reproducibility. Under optimal conditions， real-time extraction and ionization of chemical constituents from ginger tissue were achieved， yielding representative mass spectral fingerprints for each geographical origin. Multivariate statistical tools were applied to interpret the complex mass spectrometry data. Principal component analysis （PCA） provided an overview of sample distribution and revealed inherent clustering trends according to origin. Partial least squares discriminant analysis （PLS-DA） further improved the classification accuracy by filtering out unrelated variations and emphasizing the ions that contributed most to inter-regional differences. Through this approach， 27 compounds were consistently detected and identified， among which three key markers， such as 6-gingerol， 8-gingerol， and L-serine， were selected as characteristic of origin-related variation. A quantitative method was developed for these three markers， demonstrating excellent linearity across a broad content range （2.0-20 000.0 μg/g） with coefficients of determination （*R*²） no less than 0.996. Sensitivity was assessed in terms of limits of detection and limits of quantification， which ranged from 3.0 μg/g to 20.0 μg/g and 10.0 μg/g to 50.0 μg/g， respectively. Recoveries varied between 98.8% and 100.9%， indicating high accuracy， while repeatability was excellent， with relative standard deviations （RSDs） no more than 1.6%. When the established method was applied to ginger samples from different regions， heatmap visualization clearly illustrated the correlation between geographic origin and the abundance of the three characteristic compounds. For instance， Yunnan samples exhibited notably higher contents of certain gingerols， whereas those from Henan were richer in L-serine. This chemical fingerprinting strategy offers a reliable and efficient means for rapid origin verification and quality assessment. In conclusion， the iEESI-MS platform developed in this study combines minimal sample preparation， rapid analysis， and high sensitivity， making it well-suited for high-throughput applications. It holds significant potential not only for ginger authentication but also for quality control of other medicinal plants， food traceability systems， and the protection of geographically indicated products. Future studies may expand the database to include more regions and varieties， further improving the robustness and general applicability of the model.

生姜为姜科植物姜*Zingiber officinale* Rosc.的新鲜根茎^［1］^。生姜因其特有的芳香气味和辛辣味而成为世界上广泛使用的食品添加剂和香料。生姜全球共有53个基因和1 300种^［2，3］^，它分布在许多热带和亚热带地区，主要的原产地是印度、中国、印度尼西亚和尼日利亚，其中中国是第二大生姜生产国。生姜在中国、韩国和日本等亚洲国家常作为药物使用，具有解表散寒，温中止呕，化痰止咳，解鱼蟹毒的功效^［1］^。生姜的有效成分（如姜辣素、氨基酸）含量和纯度直接决定其药效的强弱与安全性。因而，对其质量评价直接关系药效的强弱。而生姜的功效成分含量通常受到其产地的影响^［4］^。在中国，药用姜主要分布在四川、贵州、云南、河南、广西等地^［4］^。生姜的化学成分多而复杂，研究生姜的多种特征成分及其不同的产地差异，对实现不同产地生姜的质量评价和产地的可追溯性具有重要意义，进而可以优选药效更佳的生姜直接入药或为新药开发提供高质量的原料。

近年来，质谱法因其灵敏度高、分析速度快、样品消耗量少等优点，被广泛用于中药样品中化学成分的分析^［5-8］^。生姜中的挥发油成分主要采用气相色谱-质谱联用技术（GC-MS）进行检测^［9-11］^，而二芳基庚烷类化合物和姜辣素可采用液相色谱-质谱联用技术（LC-MS）进行分析^［12-14］^，这两种方法虽然在中药定性分析中占据中心地位，但需要复杂的样品预处理，既费时又费力，并可能导致样品中活性成分的损失。因此，开发一种无需样品预处理的快速、灵敏的内部信息采集方法非常重要。目前已有基于内部萃取电喷雾电离质谱法（iEESI-MS）对多种不同药用部位中药化学成分分析研究，如种子类柏子仁^［15］^和酸枣仁^［16］^，根和根茎类地黄^［8］^、三七^［17］^、人参^［18］^，果实类枳壳^［19］^。该方法无需样品预处理即可直接获取中药组织样品的内部信息，具有原位分析、高速、灵敏度高等优点，从而避免了成分信息丢失，实现了快速、实时分析的优势。但不同中药含有的化学成分差异性大，且生姜含有的化学成分种类、含量及分布、宏观形态及微观的细胞排列等与现有已发表的中药存在较大差异，导致现有方法无法直接运用于生姜的质量评价，急需开发一种用于生姜的直接质谱分析方法，用于其不同产地间的质量评价。

根据中国地理标志产品的分布情况，本研究选用四川犍为姜、云南罗平小黄姜、贵州水城小黄姜和河南怀姜进行研究。开发一种内部萃取电喷雾电离质谱法，方便且快速地对不同产地生姜进行多指标质量评价。为了进一步表征和可视化4个产地的生姜样品之间的差异，建立了主成分分析（PCA）和偏最小二乘法判别分析（PLS-DA）模型来证明4个产地样品之间化学成分的差异，还揭示了生姜的化学成分与特定产地之间的相关性。

## 1 实验部分

### 1.1 仪器、试剂与材料

iEESI电离源本实验室自制（萃取腔体积约10 mL，喷雾针类型为金属毛细管），LTQ-XL型线性离子阱质谱仪（美国Thermo Scientific公司），微量注射泵（保定兰格恒流泵有限公司），FA1004B型电子天平（上海精密科学仪器有限公司），Smart-DUVF综合型超纯水机（上海和泰仪器有限公司）。针头式滤器（尼龙66，0.22 μm，13 mm，天津津腾实验设备有限公司），双圈定性滤纸（Φ7 cm，杭州思拓凡生物科技有限公司），Eppendorf微量移液器（2~20 µL）。

甲醇（色谱纯）购自德国默克公司，L-丝氨酸（纯度98%）购自北京索莱宝科技有限公司，6-姜酚（纯度98%）、8-姜酚（纯度98%）购自中国上海源叶生物科技有限公司。

四川、云南、贵州和河南的生姜药材购自江西省南昌市心诚大药房，经中药师鉴定为姜科植物姜*Zingiber officinale* Rosc.的新鲜根茎。

### 1.2 标准溶液的配制

分别称取5.0 mg L-丝氨酸、6-姜酚和8-姜酚标准品，向其中加入1.0 mL 80%（体积分数）甲醇水溶液，配制成5.0 mg/mL的标准溶液。按照梯度稀释法，分别将3种化学成分的标准溶液依次稀释至不同浓度，详见表1。

**表1:** 3种化学成分标准溶液的质量浓度

Standard substance	Mass concentrations/（μg/mL）
L-Serine	1.0， 10.0， 100.0， 1000.0， 2000.0， 5000.0
6-Gingerol	1.0， 10.0， 100.0， 500.0， 1000.0， 2000.0
8-Gingerol	0.5， 1.0， 10.0， 50.0， 100.0， 500.0

### 1.3 生姜成分的iEESI-MS分析

精确称取5.0 mg生姜样品，经碾碎后以滤纸包裹，并置于样品仓中。在进样针端施加4.5 kV高压，随后以12 μL/min的流速将80%甲醇水溶液作为萃取剂经毛细管注入样品仓，实现对生姜中有效成分的萃取，以供后续分析。内部萃取电喷雾电离源示意图见图1。

**图1:**
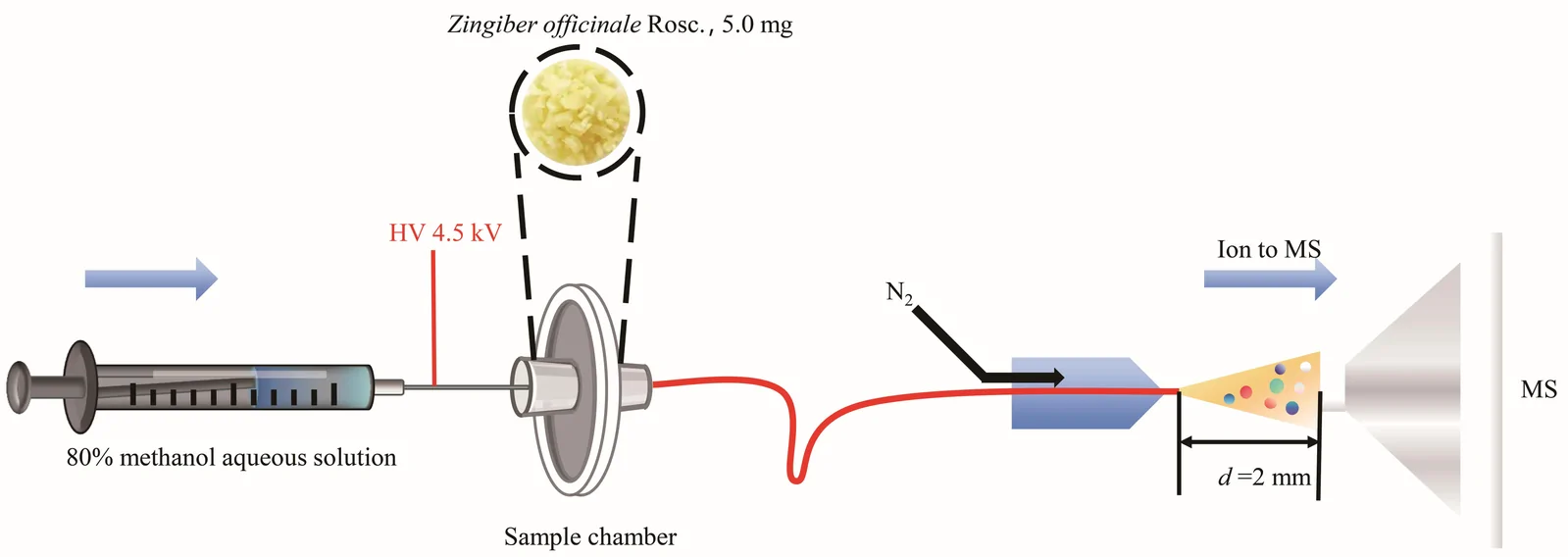
内部萃取电喷雾电离源示意图 HV： high voltage； *d*： distance from ion source nozzle to mass spectrometer （MS）.

质谱条件：正离子检测模式；质量扫描范围*m/z* 50~500；电离电压4.5 kV；离子传输管温度200 ℃；毛细管电压30 V；透镜电压110 V；电喷雾溶剂流速12 μL/min；雾化气（N_2_，纯度99.999%）压强0.5 MPa。在进行串联质谱检测时，母离子隔离宽度为1.0~1.2 u，碰撞能量20%~30%，活化值*Q*为0.25，碰撞时间30 ms。其他检测参数由LTQ-Tune系统自动优化。

## 2 结果与讨论

### 2.1 质谱条件优化

本研究采用iEESI-MS对生姜样品进行分析，质谱数据显示系统进样稳定，表明该方法适用于生姜基质中化学成分的检测。为进一步提升iEESI-MS对生姜样品的分析性能，本文以6-姜酚的二级定量离子信号为评价指标，以其3次测量的平均信号强度作为优化依据，系统考察了萃取剂、离子源喷雾口到质谱口的距离、喷雾电压、离子传输管温度等关键参数，从而实现分析条件的优化。

#### 2.1.1 萃取剂的优化

萃取剂在实验中兼具提取目标成分与电喷雾电离溶剂的双重功能，其组成比例对目标化合物的萃取效率与电离响应具有显著影响。为系统评估萃取剂组成对分析性能的影响，本研究以6-姜酚的二级质谱信号强度为评价指标，比较了甲醇体积分数分别为0、20%、40%、60%、80%及100%的甲醇水溶液的检测效果。结果如图2a所示，随着甲醇比例的增加，6-姜酚目标离子的信号强度呈现先上升后下降的趋势。在甲醇体积分数为80%时，信号强度达到最大值，表明该条件下萃取效果最佳。因此，最终确定选用80%甲醇水溶液作为萃取剂。

**图2:**
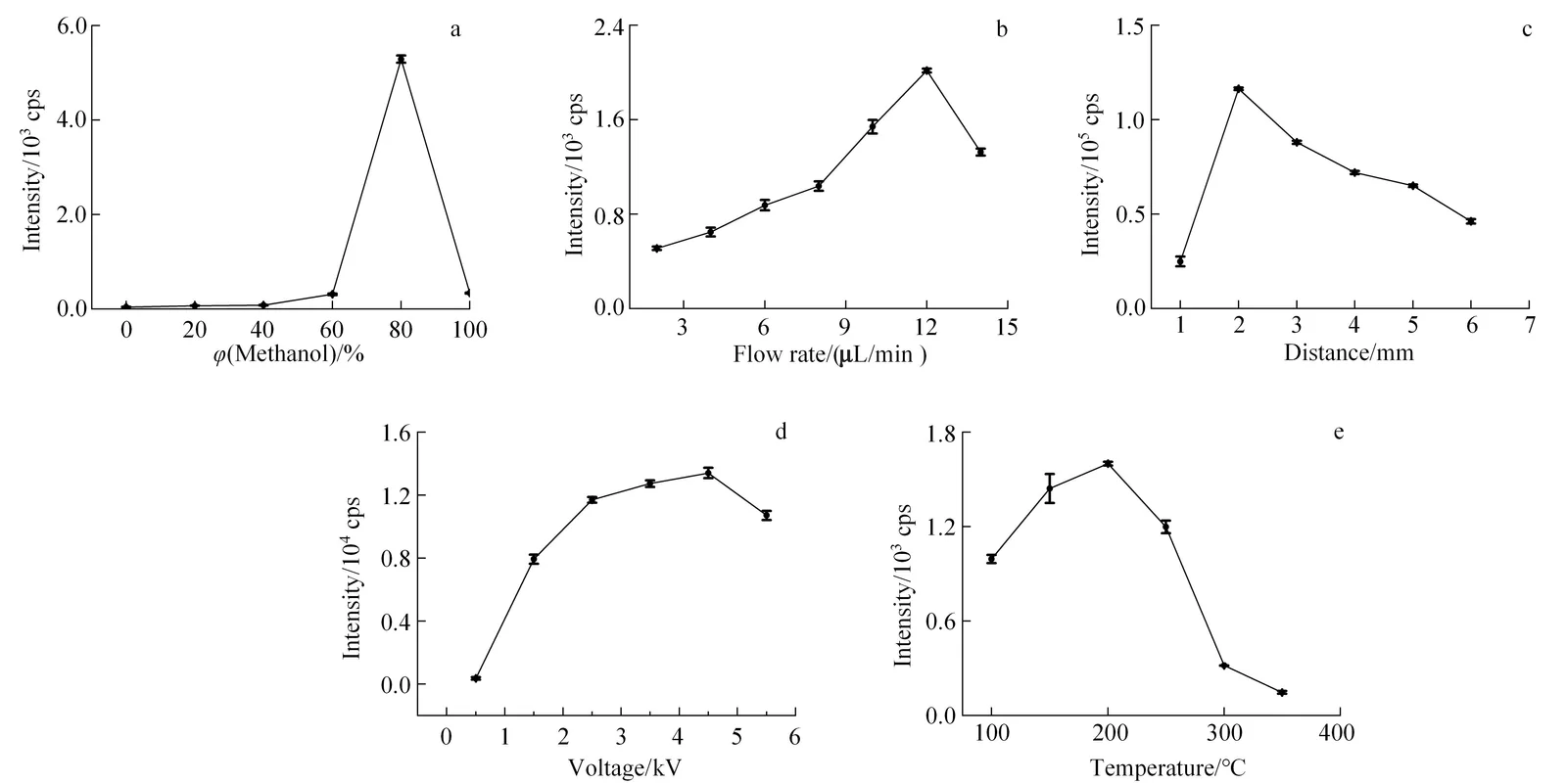
不同实验条件对信号强度的影响（*n*=3） a. volume fraction of methanol in methanol aqueous solution； b. flow rate of extraction agent； c. distance from ion source nozzle to mass spectrometer； d. ionspray voltage； e. capillary temperature.

#### 2.1.2 萃取剂流速的优化

本研究系统考察了流速在2~14 µL/min范围内（分别为2、4、6、8、10、12和14 µL/min）对6-姜酚质谱信号强度的影响。结果如图2b所示，随着流速从2 µL/min升高至12 µL/min，目标离子信号强度逐步增强；然而，当流速进一步增至14 µL/min时，信号强度显著下降，推测是流速过高导致电离效率降低。因此，为获得最佳的萃取和电离效率，确定选用12 µL/min作为后续实验的萃取剂流速。

#### 2.1.3 离子源喷雾口到质谱口的距离

本研究考察了离子源喷雾口到质谱口的距离为1~6 mm时对目标信号强度的影响，结果示于图2c。可见，信号强度呈先升高后降低的趋势，当离子源喷雾口到质谱口的距离大于2 mm时，进入质谱口中的喷雾减少，信号强度降低。因此，选择离子源喷雾口到质谱口的距离为2 mm。

#### 2.1.4 喷雾电压的优化

本研究考察了喷雾电压在0.5~5.5 kV范围内对6-姜酚信号强度的影响。结果如图2d所示，随着电压从0.5 kV升至4.5 kV，信号强度逐渐增强，表明较高的电压有助于提升电离效率；然而，当电压进一步升高至5.5 kV时，信号强度出现下降，这可能源于电压过高引发的电晕放电现象导致空气中与喷雾针尖极性相反的离子与目标离子发生中和反应，从而降低目标离子的传输效率。基于上述结果，最终选定4.5 kV作为优化后的喷雾电压。

#### 2.1.5 离子传输管温度的优化

本研究系统评估了传输管温度在100~350 ℃范围内对6-姜酚信号强度的影响。结果如图2e所示，随着温度从100 ℃升至200 ℃，信号强度逐渐增强并在200 ℃时达到最大值；继续升高温度则导致信号下降，推测是由于高温条件下6-姜酚发生热解离所致。因此确定离子传输管的优化温度为200 ℃。

### 2.2 生姜有效成分分析

在最优条件下对生姜样品进行有效成分的iEESI-MS分析，进一步结合碰撞诱导解离（CID）技术获取碎片离子信息，通过与NIST数据库、相关文献及标准品对比，初步鉴定出生姜中27种化学成分，结果列于表2。其中，挥发油类、姜辣素类以及氨基酸类物质是生姜中主要的化学成分，例如，可观测到*m/z* 106、317、323离子峰，分别为L-丝氨酸、6-姜酚和8-姜酚的准分子离子峰，为避免假阳性结果，对*m/z* 106、317、323进行碰撞诱导解离分析，可观测到*m/z* 60、217、305特征离子碎片，如图3所示，与标准品一致。其中，*m/z* 60是L-丝氨酸（［M+H］^+^ *m/z* 106）脱去1分子甲酸（HCOOH）而形成，*m/z* 217可以用6-姜酚（［M+Na］^+^ *m/z* 317）麦克拉弗蒂重排和中性烷基部分的丢失来解释，*m/z* 305是8-姜酚（［M+H］^+^ *m/z* 323）丢失1分子水（H_2_O）而形成，表明该方法可对生姜中的主要化学组分进行有效区分。

**表2:** 生姜中鉴定出的化学成分

No.	Compound	Molecular formula	Ion form	Parent ion（*m/z*）	Fragment ions （*m/z*）	Refs.
1	carveol （香芹醇）	C_10_H_16_O	［M+H］^+^	153	135， 93	［^20^］， NIST
2	safrole （黄樟素）^*^	C_10_H_10_O_2_	［M+H］^+^	163	147， 133， 131， 103	［^21^］， NIST
3	sinapyl alcohol （芥子醇）	C_11_H_14_O_4_	［M+H-H_2_O］^+^	193	161， 133， 105	［^22^］， NIST
4	6-gingerol （6-姜酚）^*^	C_17_H_26_O_4_	［M+Na］^+^	317	217	［^23^］， standard
5	diacetoxy-6-gingerdiol （二乙酸氧基-6-姜醇）^*^	C_21_H_32_O_6_	［M+Na］^+^	403	343， 163， 261， 137， 321， 283	［^23^］
6	methyl diacetoxy-6-gingerdiol （甲基二乙氧基-6-姜二醇）	C_22_H_34_O_6_	［M+Na］^+^	417	357， 177， 275， 151， 297， 335	［^23^］
7	8-gingerol （8-姜酚）^*^	C_19_H_30_O_4_	［M+H］^+^	323	305， 291	［^23^］， standard
8	methyl-8-gingerol （甲基-8-姜酚）	C_20_H_32_O_4_	［M+H］^+^	337	319， 199	［^23^］
9	acetoxy-10-gingerol （乙酰氧基-10-姜酚）^*^	C_24_H_38_O_4_	［M+H］^+^	391	331， 373	［^23^］
10	8-shogaol （8-姜烯酚）	C_19_H_28_O_3_	［M+H］^+^	305	137	［^23^］， NIST
11	12-shogaol （12-姜烯酚）^*^	C_23_H_36_O_3_	［M+H］^+^	361	137	［^23^］
12	1-dehydro-6-gingerdione （1-脱氢-6-姜酮）	C_17_H_22_O_4_	［M+H］^+^	291	273， 191， 177	［^23^］
13	1-dehydro-10-gingerdione （1-脱氢-10-姜酮）^*^	C_21_H_30_O_4_	［M+H］^+^	347	329， 177， 137	［^23^］
14	3，5-dihydroxy-1，7-bis（4-hydroxy-3，5-dimethoxyphenyl） ［3，5-二羟基-1，7-二（4-羟基-3，5-二甲氧基苯基）］	C_23_H_32_O_8_	［M+H］^+^	437	401， 419， 193	［^24^］
15	L-histidinol （L-组氨醇）^*^	C_6_H_11_N_3_O	［M+H］^+^	142	124， 60， 81， 95， 107	［^25^］， NIST
16	L-serine （L-丝氨酸）^*^	C_3_H_7_NO_3_	［M+H］^+^	106	88， 60	［^25^］， standard
17	L-proline （L-脯氨酸）^*^	C_5_H_9_NO_2_	［M+H］^+^	116	98， 70	［^25^］， NIST
18	L-aspartic acid （L-天冬氨酸）^*^	C_4_H_7_NO_4_	［M+Na］^+^	156	110， 139， 95， 83	［^25^］， NIST
19	asparagine （天冬酰胺）^*^	C_4_H_8_N_2_O_3_	［M+Na］^+^	155	138， 110， 66， 95	NIST
20	choline （胆碱）^*^	C_5_H_14_NO	M^+^	104	60， 45， 58	［^26^］， NIST
21	*trans*-2，4-dimethoxycinnamic acid（反-2，4-二甲氧基肉桂酸）^*^	C_11_H_12_O_4_	［M+H］^+^	209	191， 167， 131	NIST
22	maltose （麦芽糖）^*^	C_12_H_22_O_11_	［M+Na］^+^	365	203， 185， 305， 347	NIST
23	3-hydroxydecanoic acid （3-羟基癸酸）^*^	C_10_H_20_O_3_	［M+H-H_2_O］^+^	171	143， 153， 125， 156， 154	NIST
24	3-indoleacrylic acid （3-吲哚丙烯酸）^*^	C_11_H_9_NO_2_	［M+H-H_2_O］^+^	170	142， 124， 115， 95	NIST
25	3-ureidopropionic acid （3-酰脲丙酸）^*^	C_4_H_8_N_2_O_3_	［M+H-H_2_O］^+^	115	70， 98， 72， 73， 87， 61， 56， 44	NIST
26	*β*-cyano-L-alanine （*β*-氰基-L-丙氨酸）^*^	C_4_H_6_N_2_O_2_	［M+H］^+^	115	74， 69	NIST
27	uracil （尿嘧啶）^*^	C_4_H_4_N_2_O_2_	［M+H］^+^	113	95， 70	NIST

* Substances of variable importance in the projection （VIP） >1 of ginger from different origins. NIST： National Institute of Standards and Technology.

**图3:**
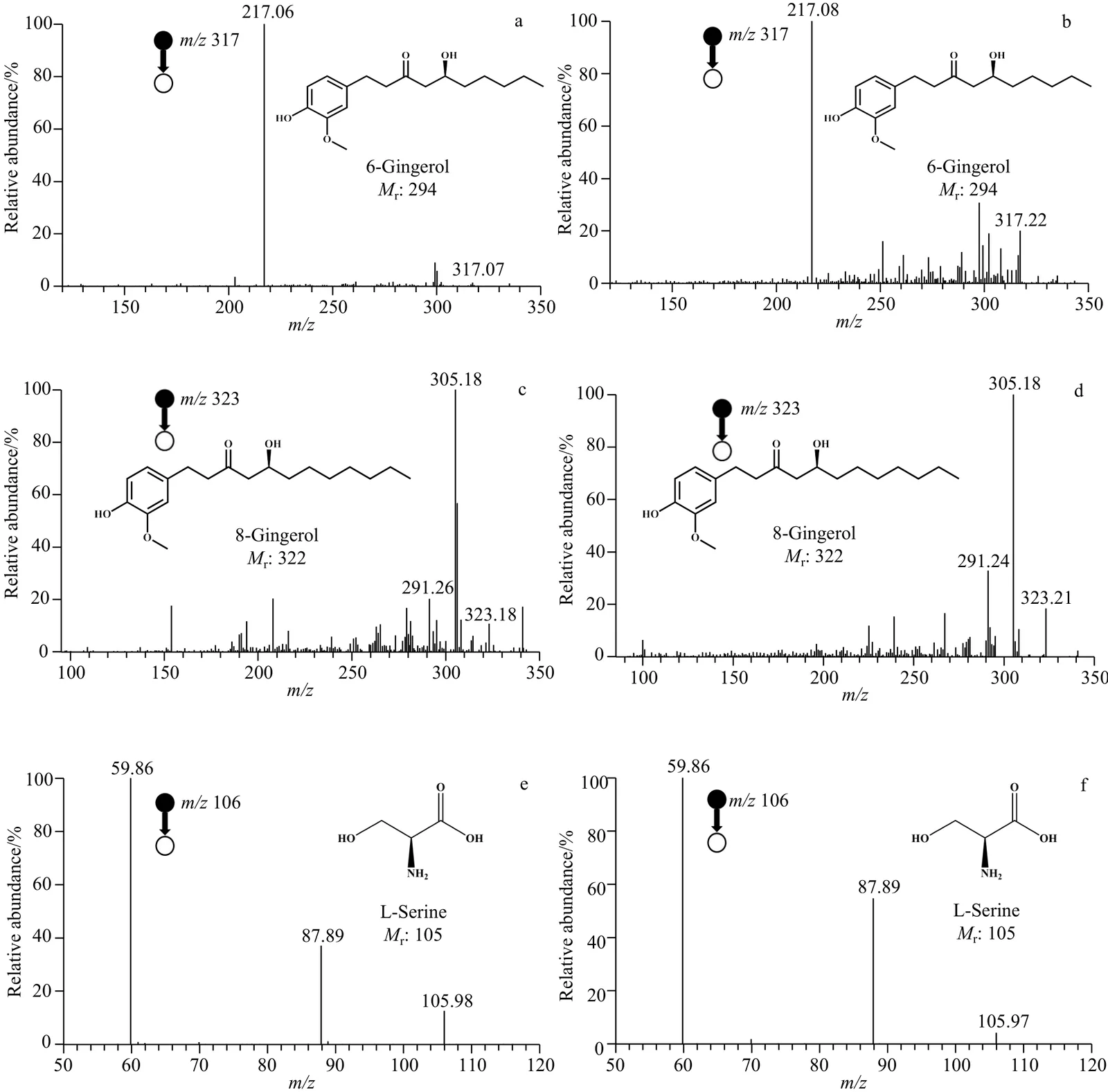
正离子模式下3种化学成分样品和标准品的二级质谱图 a. 6-gingerol sample； b. 6-gingerol standard； c. 8-gingerol sample； d. 8-gingerol standard； e. L-serine sample； f. L-serine standard.

### 2.3 不同产地样品分析

不同产地生姜的iEESI-MS指纹图谱见图4。iEESI-MS技术能有效获取生姜化学成分信号的信息，这些成分一般以［M+H］^+^形式存在，但生姜样品中含有丰富的无机离子（如Na^+^、K^+^）等，在iEESI离子化过程中会参与电离反应，使生姜样品中的物质电离得到多种形态的离子化产物，如6-姜酚的［M+K］^+^ *m/z* 333和［M+Na］^+^ *m/z* 317。进一步对比谱图发现，虽然主要的物质信号相似，如*m/z* 104、175、219、274、333、491等，但不同产地的信号强度存在明显变化，如云南的6-姜酚（［M+K］^+^ *m/z* 333和［M+Na］^+^ *m/z* 317）和胆碱（M^+^ *m/z* 104）等成分信号均高于其他产地。

**图4:**
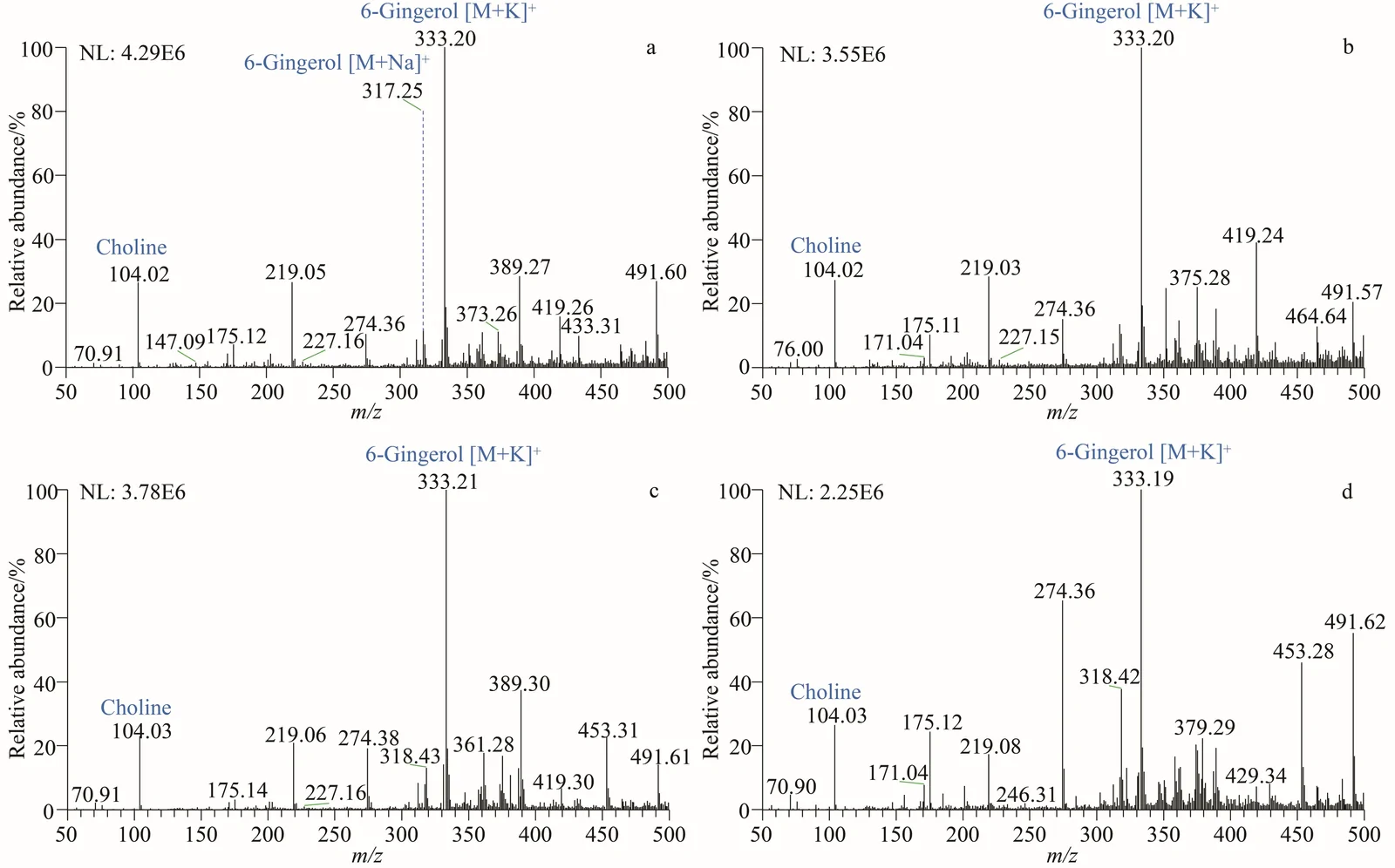
不同产地生姜的一级质谱图 a. Yunnan Province； b. Guizhou Province； c. Sichuan Province； d. Henan Province. NL： normalized intensity.

### 2.4 统计分析

#### 2.4.1 不同产地生姜主要成分的PCA、PLS-DA分析

为了分析不同产地生姜的化学成分差异，将得到的iEESI-MS一级质谱数据按要求用MATLABR2016a软件处理后，再导入SIMCA14.1软件进行PCA分析，结果示于图5。可以发现，不同产地生姜被明显分成4组，说明其化学成分有显著差异。为找到不同产地生姜成分的主要差别，采用PLS-DA考察变量重要性投影（VIP）值，VIP值越大表明该变量对模型越重要，通常将VIP>1的变量视为该模型的重要标记物。首先构建了这4种产地生姜的PLS-DA模型，*R* ^2^ *Y*=0.989，*Q* ^2^ =0.988，*R* ^2^>*Q* ^2^>0.5，说明建立的PLS-DA模型具有极强的解释能力及预测能力（图5），可以可靠地用于区分不同组别的样本，且置换检验结果显示，*R* ^2^截距为0.097 7，小于0.3，*Q* ^2^截距为-0.198，小于0.05，说明本模型没有过拟合，可用于考察变量VIP值（图6）。接着筛选出20个VIP>1的化合物，结果列于表2。在这20个标记的化合物中，有6个是姜辣素类化合物，表明其是区分不同产地生姜的重要标志性化合物。

**图5:**
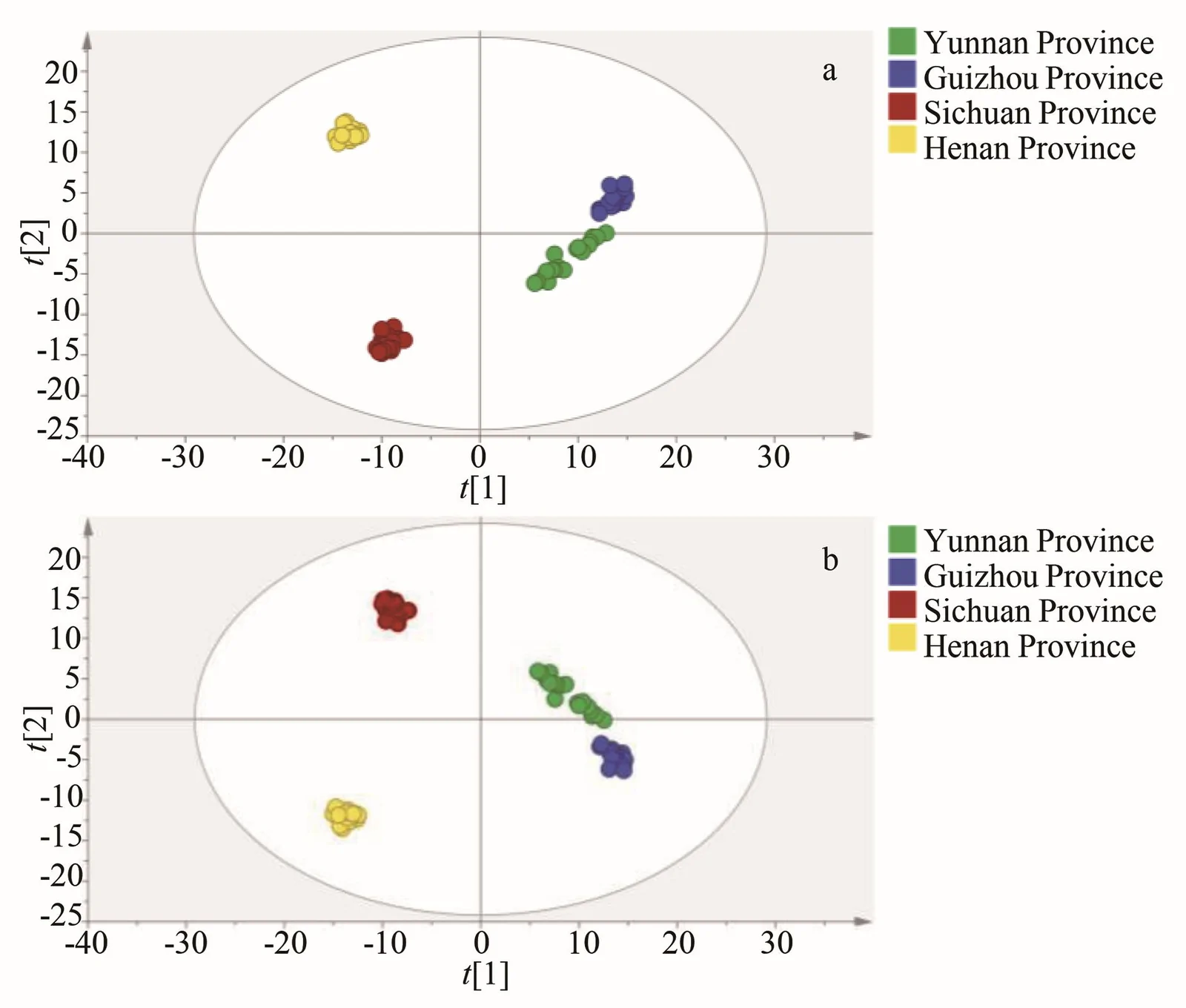
不同产地生姜样品的（a）PCA和（b）PLS-DA图

**图6:**
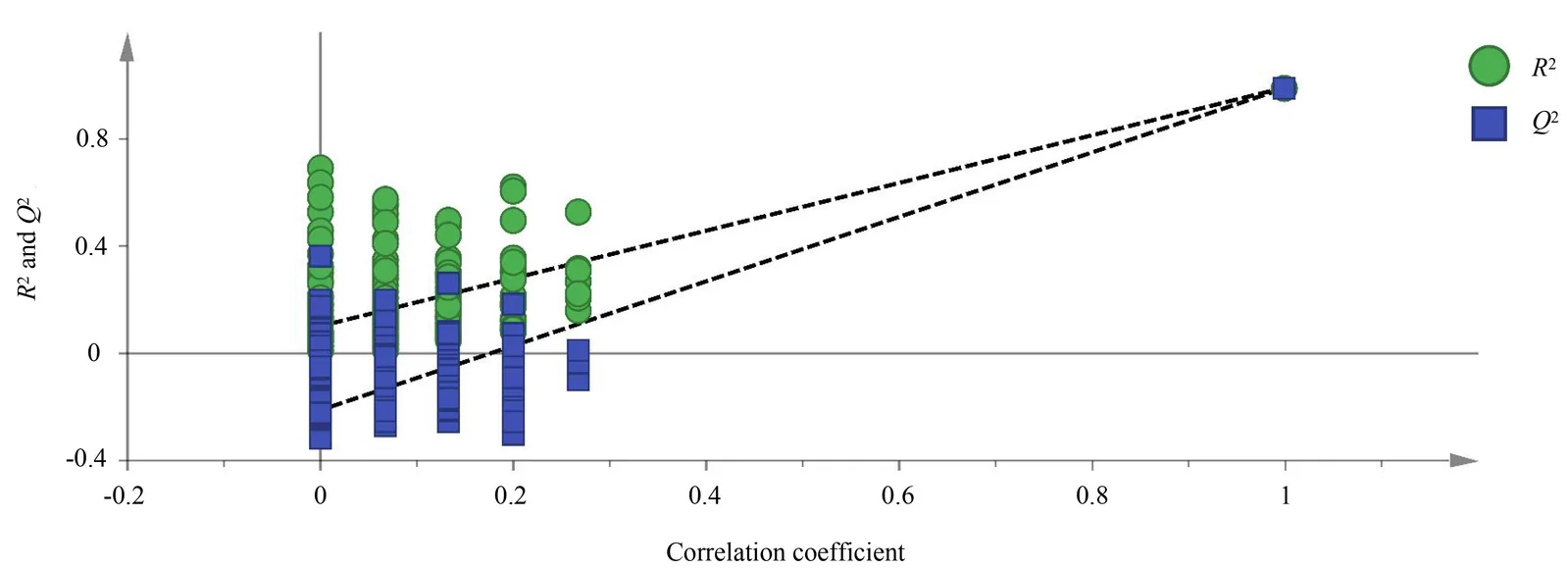
不同产地生姜样品PLS-DA的置换检验图

#### 2.4.2 生姜主要成分与差异成分的相关性分析

图7为生姜主要成分与主要差异成分的相关性热图。根据相关性热图可知，6-姜酚与L-丝氨酸呈现显著正相关，说明L-丝氨酸可能直接或间接地参与了6-姜酚的生物合成可能性分析，即L-丝氨酸含量高的样本，可能更有利于6-姜酚的合成。8-姜酚与L-丝氨酸则呈现显著的负相关，这些成分之间的强关联，使它们可作为生姜产地的特征标志物，用于认证生姜品质。这种关联规律也可为生姜种植调控、加工优化、产品开发提供精准靶点，也为其他中药的代谢组学研究提供了依据。

**图7:**
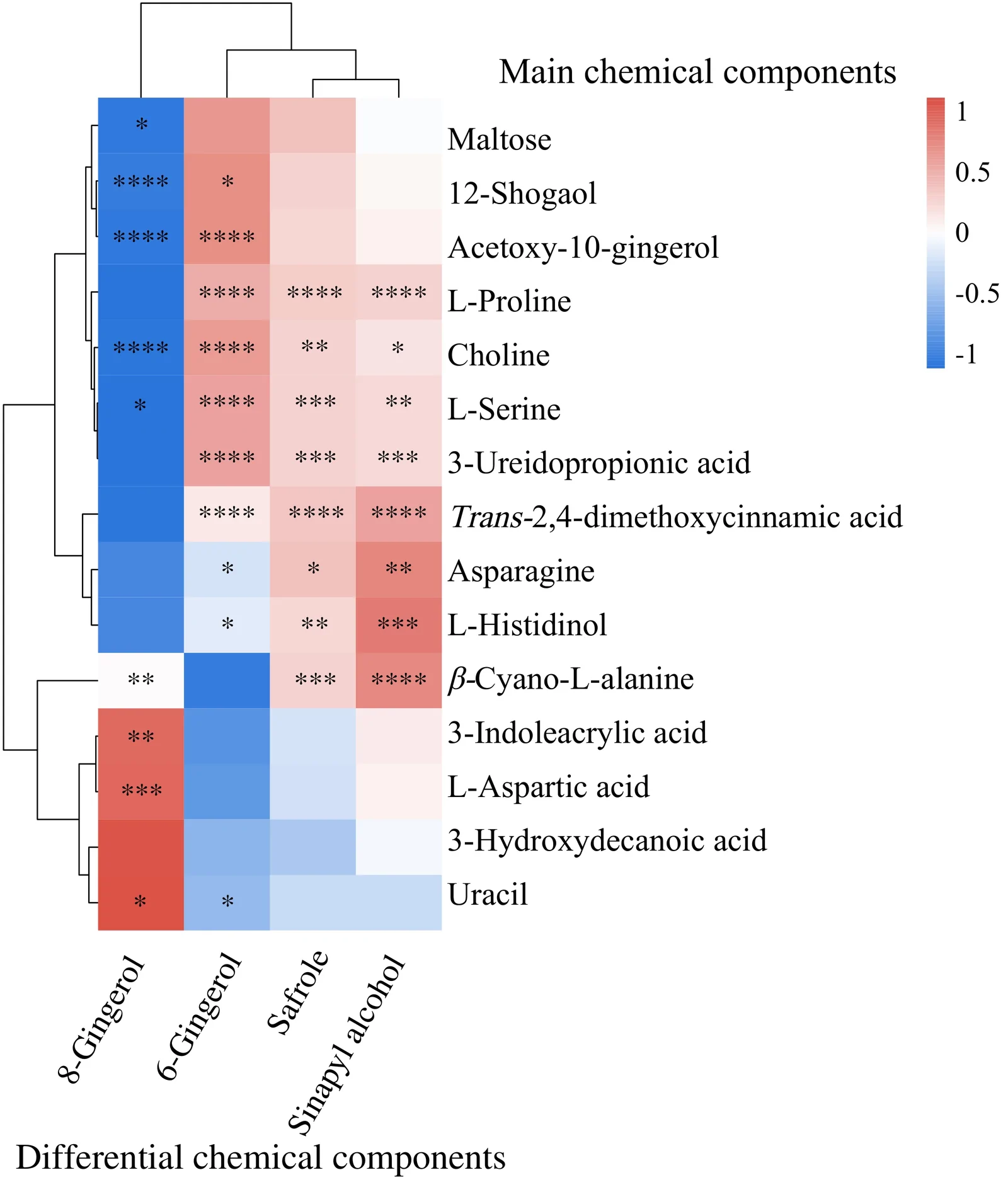
生姜主要成分和差异成分的相关性热图

#### 2.4.3 不同产地生姜的热图分析

为了进一步说明这3种化学成分在不同产地之间的变化，选用云南、四川、贵州和河南这4个不同产地的生姜一级质谱信号进行热图分析，结果示于图8。热图显示，云南生姜6-姜酚和L-丝氨酸含量显著高于其他产地生姜，河南生姜8-姜酚含量最高，反映了产地环境（气候、土壤、栽培模式等）对生姜化学成分含量的影响，云南普遍海拔较高，紫外线辐射强，昼夜温差大。云南高原日照时间长，受印度洋爬升气流的影响，降水量相对较高^［27］^。高原高强度紫外线和适度逆境胁迫（如温差）会显著激发植物的次生代谢途径，作为防御机制。这很可能是云南生姜6-姜酚含量高的主要环境原因。L-丝氨酸作为初生代谢产物，其积累也可能与高海拔环境下特殊的氮代谢有关。河南具有日照充足、气温适宜与昼夜温差大等特点。然而，其春季降水较少，干热风天气频发，同时降水分布不均，呈现出明显的雨热同期特征^［28］^。这样的气候条件可能恰好最适合当地品种合成8-姜酚。贵州省地形和气候特殊，是典型的喀斯特地貌，气候上属于亚热带润湿季风气候，雨量丰富，湿度大^［29］^。特定的温湿度条件可能影响了姜中酶的活性，促使6-姜酚与8-姜酚的积累，反映了其品质与产地存在较大的关联性。

**图8:**
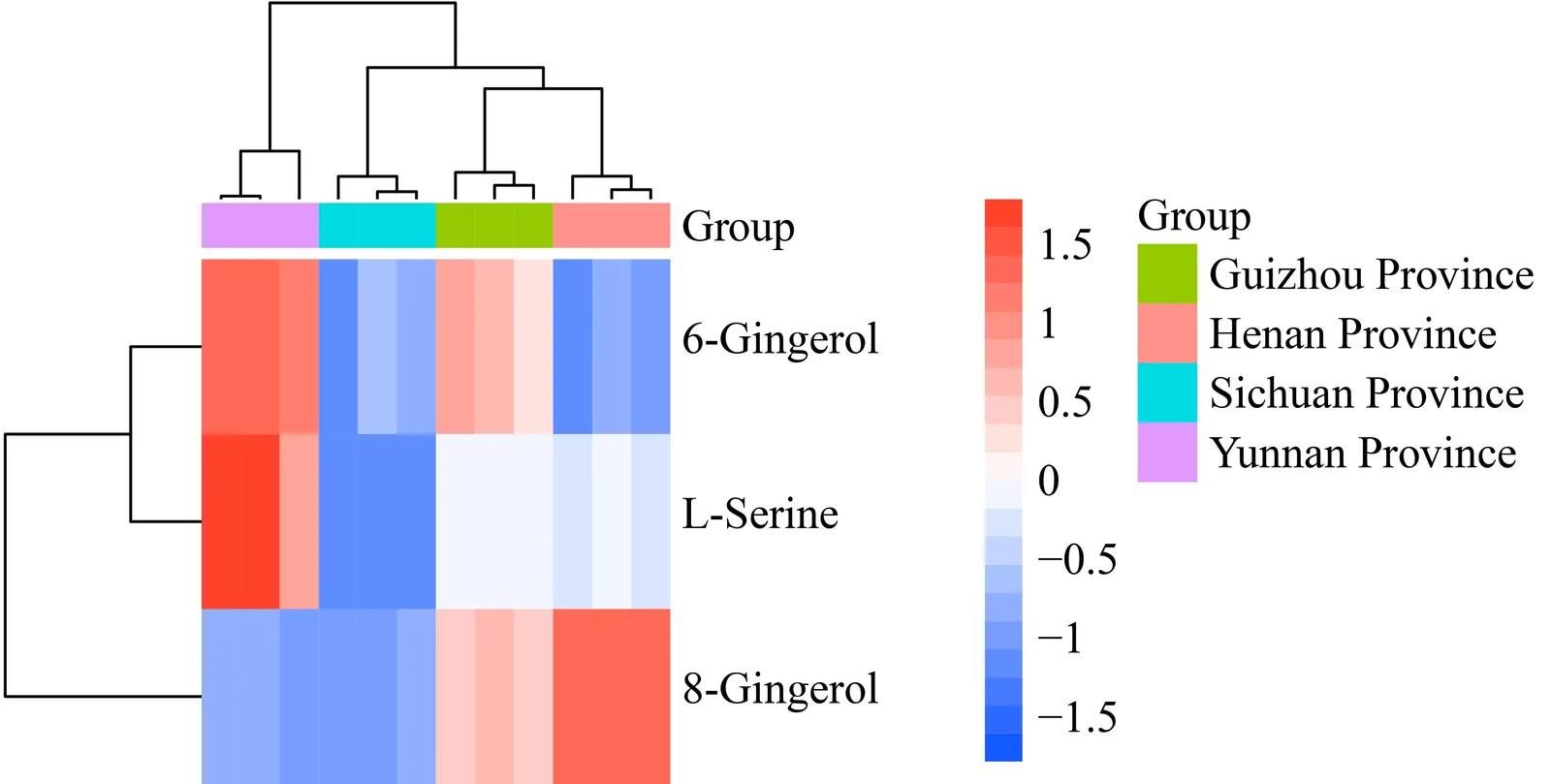
不同产地生姜的热图

### 2.5 方法学考察

#### 2.5.1 线性关系、检出限和定量限

统计学分析结果显示，6-姜酚、8-姜酚和L-丝氨酸为区分不同产地生姜的主要成分。因而我们对这3种成分进行方法学考察。按1.2节方法配制L-丝氨酸、6-姜酚和8-姜酚标准品溶液，以云南产地的生姜为样品，于样品上分别滴加不同浓度的标准溶液20.0 μL，采用iEESI-MS进样测定，并且选择6-姜酚、8-姜酚和L-丝氨酸的二级特征碎片离子作为定量离子按1.3节质谱条件进行分析。以上述化学成分含量为横坐标，以目标离子的3次平均质谱信号强度为纵坐标绘制定量曲线，通过决定系数（*R* ^2^）来评估标准曲线的线性，以信噪比≥3和10的含量分别确定分析方法的检出限（LOD）和定量限（LOQ）。结果表明，L-丝氨酸、6-姜酚和8-姜酚在各自的线性范围内具有良好的线性关系，*R* ^2^值均在0.996~0.999范围内，说明iEESI-MS分析方法可以满足同时检测生姜中3种化学成分的要求（见表3）。

**表3:** 生姜中3种化学成分的线性范围、回归方程、决定系数、检出限和定量限

Sample	Linear range /（μg/g）	Regression equation	*R* ^2^	LOD/（μg/g）	LOQ/（μg/g）
L-Serine	4.0-20000.0	*y*=345.75*x*+202.59	0.997	20.0	50.0
6-Gingerol	4.0-4000.0	*y*=3661.7*x*+478.98	0.996	10.0	30.0
8-Gingerol	2.0-2000.0	*y*=46.977*x*+4.0563	0.999	3.0	10.0

*y*： signal intensity of characteristic MS/MS fragment ion； *x*： content， μg/g．

#### 2.5.2 加标回收率和重复性

称取5.0 mg生姜样品，用滤纸包裹后置于样品仓中，随后分别加入20.0 µL不同浓度的标准溶液，以此计算生姜中3种化学成分各自的3个加标水平，如下：L-丝氨酸800、4 000、20 000 μg/g；6-姜酚400、1 000、2 500 μg/g，8-姜酚100、200、400 μg/g。按照第1.3节方法进行测定，得到3种化学成分不同加标水平下的回收率，结果见表4。3种化学成分的平均回收率均为98.8%~100.9%。同一加标量的样品3次测量的相对标准偏差（RSD）均不大于1.6%。实验结果表明，iEESI-MS分析方法在本实验中有着良好的精密度和重复性。

**表4:** 3种化学成分在生姜中3个水平下的加标回收率（*n*=3）

Compound	Background/（μg/g）	Added/（μg/g）	Found/（μg/g）	Recovery/%	RSD/%
L-Serine	4245.6	800.0	5044.9	99.9	1.6
		4000.0	8281.6	100.9	1.4
		20000.0	24375.0	100.6	0.2
6-Gingerol	920.7	400.0	1315.9	98.8	1.2
		1000.0	1917.7	99.7	0.5
		2500.0	3434.1	100.5	0.5
8-Gingerol	239.9	100.0	340.0	100.1	1.0
		200.0	441.2	100.7	0.4
		400.0	640.8	100.2	1.0

#### 2.5.3 实际样品中化学成分检测

为了获得生姜样品中化学成分的定量信息，采用iEESI-MS技术直接测定云南、四川、贵州和河南4个不同产地的3种化学成分（L-丝氨酸、6-姜酚和8-姜酚）的含量，结果见表5。

**表5:** 不同产地实际样品的分析结果（*n*=3）

Origin	L-Serine		6-Gingerol		8-Gingerol	
	Content/（μg/g）	RSD/%	Content/（μg/g）	RSD/%	Content/（μg/g）	RSD/%
Yunnan Province	4336.2	0.8	945.2	1.0	176.9	4.8
Sichuan Province	529.3	2.1	749.3	2.9	95.6	3.0
Guizhou Province	2418.7	0.3	864.1	5.8	208.8	0.5
Henan Province	2016.0	1.1	696.6	2.4	230.0	0.1

#### 2.5.4 与报道方法比较

与现有文献相比（见表6），本方法不需要样品预处理且需要的样品量和时间远低于文献报道，说明本研究建立的分析方法更方便快捷。

**表6:** 3种基于质谱的生姜样品化学成分分析方法

Method	Sample pretreatment	Volume of solvent/mL	Sample quality/mg	Time/min	Ref.
GC-MS	drying and grinding	/	100.0	~11.5	［^30^］
LC-MS	grinding， extraction， centrifugation and filtration	~20.0	5000.0	~30.0	［^31^］
iEESI-MS	/	~0.1	5.0	~0.5	this work

iEESI-MS： internal extractive electrospray ionization mass spectrometry.

## 3 结论

本工作建立了iEESI-MS结合统计分析技术的高通量化合物鉴别方法，用于生姜样品分析。新鲜的生姜样品能够以高通量和快速的方式检测，无需样品预处理。从生姜中初步鉴定出27种化学成分，包括挥发油、姜辣素和其他化合物。基于iEESI-MS结果，PLS-DA方法为区分生姜的产地提供了稳定可靠的鉴别模型。相关性分析及热图分析进一步揭示了产地与主要差异成分含量之间的关联性，并建立了主要差异成分L-丝氨酸、6-姜酚和8-姜酚的iEESI-MS定量分析方法，这些结果表明，该方法在传统中药的质量评价及产地溯源方面具有巨大的应用潜力。

## References

[1] Chinese Pharmacopoeia Commission . Pharmacopoeia of the People’s Republic of China， Part 1. Beijing： China Medical Science Press， 2020： 15， 104

[2] Kress W J， Prince L M， Williams K J . Am J Bot， 2002， 89（10）： 1682 21665595 10.3732/ajb.89.10.1682

[3] Prasath D， Karthika R， Habeeba N T， et al . PLoS One， 2014， 9（6）： e99731 24940878 10.1371/journal.pone.0099731 PMC4062433

[4] Yoon D， Choi B R， Kim H G， et al . J Food Sci， 2024， 89（11）： 7452 39390639 10.1111/1750-3841.17456

[5] Yang L， Xue Z， Li Z， et al . Phytochem Anal， 2025， 36（1）： 194 39108034 10.1002/pca.3430

[6] Du J， Li X， Tian W， et al . Drug Test Anal， 2024， 16（12）： 1537 38440922 10.1002/dta.3672

[7] Hu Y， Mei X， Cui Y， et al . Int J Mol Sci， 2025， 26（22）：11034 41303520 10.3390/ijms262211034 PMC12652469

[8] Xu J， Yu Z， Li T， et al . J Am Soc Mass Spectrom， 2023， 34（7）： 1342 37294877 10.1021/jasms.3c00043

[9] Tarfaoui K， Brhadda N， Ziri R， et al . Plants （Basel）， 2022， 11（11）： 1487 35684260 10.3390/plants11111487 PMC9182767

[10] Fayed A M， S N H， Samy W， et al . Asian Pac J Cancer Prev， 2024， 25（11）： 3895 39611913 10.31557/APJCP.2024.25.11.3895 PMC11996100

[11] Spyrou A， Batista M G F， Corazza M L， et al . Molecules， 2024， 29（4）： 871 38398623 10.3390/molecules29040871 PMC10893072

[12] Li M Q， Hu X Y， Wang Y Z， et al . Rapid Commun Mass Spectrom， 2021， 35（8）： e9029 33326132 10.1002/rcm.9029

[13] Zhang W H， Liu P X， Qiu J， et al . Chinese Journal of Chromatography， 2019， 37（10）： 1105 31642290 10.3724/SP.J.1123.2019.03024

[14] Salem M A， Zayed A， Alseekh S， et al . Phytochemistry， 2021， 190： 112843 34311278 10.1016/j.phytochem.2021.112843

[15] Wang N， Xie S， Liu P， et al . Anal Methods， 2025， 17（10）：2254 39967485 10.1039/d4ay01881h

[16] Liu P， Wang N， Xie S Y， et al . Journal of Chinese Mass Spectrometry Society， 2025， 46 （4）： 503

[17] Zhang X， Chen Z Y， Qiu Z D， et al . Phytochemistry， 2022， 194： 113030 34839132 10.1016/j.phytochem.2021.113030

[18] Yuan X， Zhang X， Xu J， et al . Foods， 2023， 12（6）： 1152 36981079 10.3390/foods12061152 PMC10048038

[19] Yang F X， Zhong L Y， Chen H W， et al . Journal of Chinese Mass Spectrometry Society， 2024， 45（6）： 763

[20] Xu L J， Xue Y L， Hou W J， et al . Agricultural Technology & Equipment， 2020（4）： 4

[21] Han M， Hou X， He G Y， et al . Modern Food Science and Technology， 2021， 37（1）： 292

[22] Zhu L， Zhou W， Wang J， et al . Microbiol Res， 2025， 292： 128031 39705829 10.1016/j.micres.2024.128031

[23] Jiang H， Sólyom A M， Timmermann B N， et al . Rapid Commun Mass Spectrom， 2005， 19（20）： 2957 16189817 10.1002/rcm.2140

[24] Jiang H， Timmermann B N， Gang D R， et al . Rapid Commun Mass Spectrom， 2007， 21（4）： 509 17238228 10.1002/rcm.2858

[25] Zhou L B， Huang J M， Wang X D . Shandong Chemical Industry， 2024， 53（17）： 181

[26] Zhang H， Zhu L， Luo L， et al . J Agric Food Chem， 2013， 61（45）： 10691 24107102 10.1021/jf4032469

[27] Gui W， Wen Q， Dong W， et al . PLoS One， 2025， 20（3）： e0319420 40067844 10.1371/journal.pone.0319420 PMC11896063

[28] Sun Y S . North Sericulture， 2019， 40（3）： 43

[29] Chen Z， Hu J， Ma C， et al . China Plastics， 2025， 39（1）： 97

[30] Yu D X， Zhang X， Guo S， et al . Food Chem， 2022， 396： 133672 35872496 10.1016/j.foodchem.2022.133672

[31] Mais E， Alolga R N， Wang S L， et al . Food Chem， 2018， 240： 239 28946267 10.1016/j.foodchem.2017.07.106

